# Late post-arthroscopy hip instability. Diagnosis, treatment, and 5-year follow-up: A case report

**DOI:** 10.1016/j.ijscr.2024.109323

**Published:** 2024-01-27

**Authors:** Milán Fernando Zárate Leal, María Bautista, Alfredo Sánchez-Vergel

**Affiliations:** a Department of Orthopedics and Traumatology, Hospital Universitario Fundación Valle del Lili, Cali, Colombia; b School of Health Sciences, Universidad ICESI, Cali, Colombia

**Keywords:** Femoroacetabular impingement, Hip arthroscopy, Iatrogenic hip dislocation, Hip capsule reconstruction, Case report

## Abstract

**Introduction:**

Hip instability following arthroscopy is a rare complication with a clinical spectrum ranging from gross dislocation (macro-instability) to micro-instability, characterized by pain and limitation for daily activities. Therefore, it should be considered as a potential differential diagnosis in patients experiencing persistent pain after hip arthroscopy.

**Case presentation:**

A 41-year-old male presented with a history of anterior hip dislocation and macro-instability symptoms three years post-hip arthroscopy. Magnetic resonance imaging (MRI) revealed a disruption of the anterior hip capsule. The patient initiated physiotherapy and resumed activities, but ten months later, experienced another anterior dislocation. Pain and apprehension during external hip rotation were evident. Three-dimensional computed tomography (CT) indicated irregularities in the anterior and superior margin of the acetabulum, while MRI arthrogram showed a rupture of the anterior capsule and deficiency in the anterior hip ligaments. Open reconstruction of the anterior capsule was performed, resulting in favorable progression over the 5-year follow-up.

**Discussion:**

This case highlights post-arthroscopy hip instability with a delayed presentation, possibly linked to chronic anterior capsule deficiency and inadequate acetabular coverage. Primary capsule repair after hip arthroscopy has proven effective in reducing the occurrence of instability symptoms and reoperations.

**Conclusions:**

Post-arthroscopic hip instability may manifest immediately after surgery or several years later. Open reconstruction of the anterior capsule emerges as a successful strategy for addressing this complication, demonstrating satisfactory outcomes in a 5-year follow-up.

## Highlights

• Post-arthroscopic hip instability varies from gross dislocation to micro-instability presented as pain and limitation for daily activities.
• Increased femoral anteversion, hip dysplasia, hyperlaxity, and traumatic injuries have been described as predisposing factors for iatrogenic hip instability.
• Post-arthroscopic hip instability can be present at any time after the procedure.
• Open reconstruction of the anterior capsule with tendon allografts is an effective option for treatment of post-arthroscopic hip instability.

## 1 Introduction

Hip instability following arthroscopy is a rare complication [[1], [2], [3]]. Almost 3 % of patients require additional interventions for its treatment [4]. Female sex increased femoral anteversion, hip dysplasia, hyperlaxity, and traumatic injuries have been described as predisposing factors for iatrogenic hip instability [1,5,6].

The clinical presentation of hip instability varies from gross dislocation (macro-instability), to micro-instability presented as pain and limitation for daily activities [3]. Thus, this condition should be considered as a differential diagnosis in patients with persistent pain after hip arthroscopy.

We present the case of hip instability, with an anterior dislocation occurring 3 years after the index procedure, the diagnostic approach, the treatment with capsular reconstruction, and the long-term results. Appropriate informed consent was obtained from the patient to disclose clinical information and images. This case report has been reported in line with SCARE criteria [7].

## 2 Case report

**Fig. 1:**
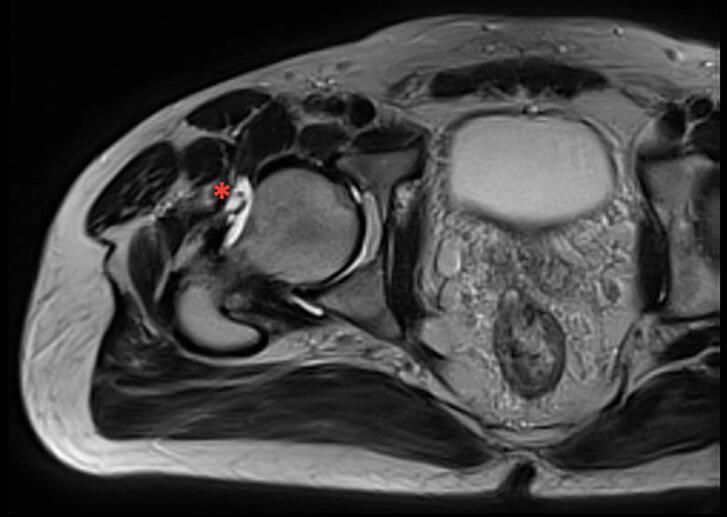
MRI of the right hip showing disruption of anterior capsular ligaments with T2 hyperintensity at the anterior aspect of the femoral head (asterisk), no additional labral tears or other abnormalities were found.

A 41 years-old male patient presented in 2013 to another hospital with left hip pain. The right hip was asymptomatic. He was diagnosed with femoroacetabular impingement and underwent simultaneous bilateral hip arthroscopy. Operative notes (other hospital) did not describe a capsular repair. He remained asymptomatic from the right hip but presented mild pain on the left. In 2016, he sustained an anterior right hip dislocation after a ground level fall on a wet floor, treated with a closed reduction at the emergency room. Later, he presented to our clinic mobilizing with crutches and complaining of groin pain and pain with hip extension; the neurovascular exam was unremarkable. Magnetic resonance imaging (MRI) evidenced a disruption of the anterior hip capsule (Fig. 1). The patient started physiotherapy with good progress and returned to his activities.

**Fig. 2:**
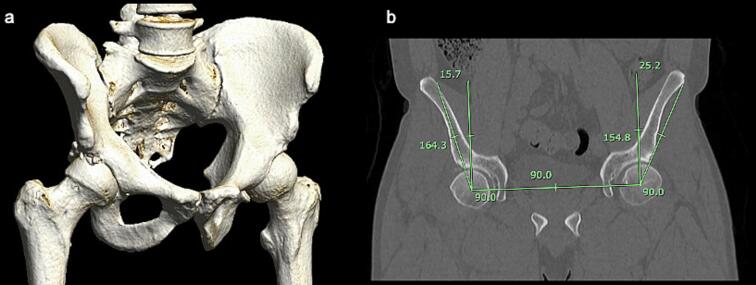
(a) CT 3D reconstruction showing irregularity in the anterior superior margin of the right acetabulum with adjacent bone fragments suggesting an apparent lack of acetabular coverage (antero-superior aspect) (b) quantification of center-edge angle at the coronal section where the irregularity of the superior-anterior margin of the acetabulum was more evident (25.2° left, 15.7° right).

**Fig. 3:**
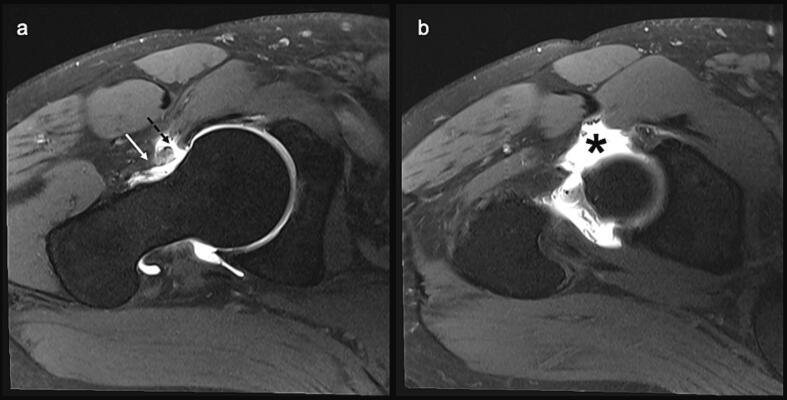
MRI arthrogram of the right hip showing (a) disruption of the joint capsule in the upper anterior region (interrupted arrow) with bulging (solid arrow) and (b) leakage of contrast to the antero-superior aspect of the hip (asterisk).

Ten months after, he sustained another anterior dislocation, while standing and pivoting on the right lower limb, forcing this hip onto external rotation, also treated with closed reduction. Pain and apprehension with the external rotation of the hip was evidenced. Three-dimensional computed tomography (CT) showed irregularity in the anterior and superior margin of the acetabulum (Fig. 2a), and the MRI arthrogram (Fig. 3) evidenced a rupture of the anterior capsule, with deficiency of the anterior ligaments of the hip.

**Fig. 4:**
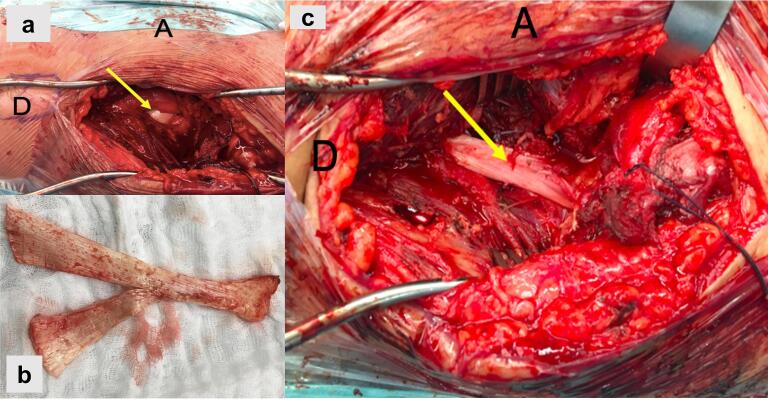
(a) Defect in the joint capsule (solid arrow) comprising the anterior and lateral aspects. (b) Achilles tendon allograft arranged in a “Y” shape to allow its anchorage in the iliac bone and proximal femur. (c) Reconstruction of the joint capsule with the Achilles tendon allograft (arrow). (D = distal, A = anterior).

Open reconstruction of the anterior capsule of the right hip was performed through direct anterior approach as described by Hueter. The anteromedial border of the capsule was dissected and separated from the psoas muscle. With the lower limb in internal rotation, the medial and lateral flaps were sutured using separate Ethibond stitches, imbricating the capsule. Intraoperatively a defect compromising the anterior and lateral aspects of the capsule were identified (Fig. 4a). The labrum was intact. An Achilles tendon allograft was arranged in a “Y” shape by completing a longitudinal cut through the tendon fibers up to half of its length (Fig. 4b). Then, it was fixed with two 5.5 mm suture anchors to the anteroinferior iliac spine, one anchor to the lesser trochanter, and 1 anchor to the greater trochanter (Fig. 4c). The patient was instructed to avoid weight-bearing for six weeks, and hip extension and external rotation for 12 weeks. The patient recovered fully, did not report any symptoms, and returned to his activities. The patient presented postoperative hypoesthesia on the lateral thigh that resolved; the lateral femorocutaneous nerve was not visualized during the procedure.

At his last visit in 2022, he described occasional pain on the right groin but no further dislocation episodes. The patient modified his activities to “protect his hip” but resumed other exercises and started jogging. The Oxford Hip Score at the last telephonic contact was 46. Clinically, there is a restricted external rotation compared to the contralateral hip.

## 3 Discussion

Several cases of anterior hip dislocation following hip arthroscopy for femoroacetabular impingement have been reported [6,8]. Nonetheless, this is the first case of post-arthroscopy hip instability with a late presentation.

In previous reports, hip dislocation, after a low-energy trauma, occurred between 0 and 4 months postoperatively. These authors described both patient and surgical factors related to instability: female gender (77.8 %), acetabular dysplasia (22.2 %), and generalized ligamentous laxity (11.1 %), unrepaired capsulotomy (77.8 %), iliopsoas release (33.3 %), labrum repair or debridement, and acetabular and femoral remodeling [1,5,6,8].

In hip arthroscopy, when a capsulotomy is performed to improve visualization and instrumentation, it is typically left unrepaired [4]; here, the iliofemoral ligament, which limits the anterior translation and external rotation of the hip, is incised [3,9]. Thus, anterior capsular defects alter these biomechanical properties and might trigger micro-instability [3,6,8]. Instability is also associated with aggressive acetabuloplasty and iliopsoas tendon release [1,6]. As hip stability depends on both static and dynamic structures, macro-instability is the result of the deficiency of both [1,10]. We hypothesize that the late presentation in our patient is due to a chronic deficiency of the anterior capsule, exacerbated by the first dislocation, and the lack of acetabular coverage (Fig. 2a and b).

The role of capsule repair after hip arthroscopy is widely studied. The systematic review and meta-analysis published by Looney et al., included 5132 hip arthroscopies, of which 3427 underwent capsule repair, demonstrated significant improvement in postoperative functional scores, regardless of the indication for arthroscopy [4]. Frank et al. compared the results of partial and complete capsule repair, and found significant improvements in functional outcomes and a lower proportion of revisions in patients undergoing a complete repair [11].

The treatment for post-arthroscopic hip instability includes open reduction, capsular plication, and iliofemoral ligament reconstruction with allografts [2,5]. We observed that open capsule reconstruction with an allograft simulating the native ligament anatomy, effectively improves pain and instability symptoms, with satisfactory long-term results.

Wylie et al., identified 33 cases of symptomatic instability in 1100 hip arthroscopies; they described that arthroscopic revision for capsule reconstruction with anchors, anterior rectus, iliotibial band grafts, or acellular dermal matrix graft also provided good functional results [10]. O'Neill et al., reported the effect of isolated capsular reconstruction in revision arthroscopy for instability in 31 patients (follow-up 3.3 years), also found improvement in functional scores and symptoms. The average time to revision of 2 years in this study might be related to the challenge of diagnosing micro-instability [9]. These symptoms must be differentiated from other conditions, including residual femoroacetabular impingement, chondro-labral tears, and acetabular dysplasia.

In conclusion, iatrogenic hip instability is an uncommon complication that can affect patients in the late postoperative period, as demonstrated by our case of hip dislocation three years after the index procedure, with open reconstruction as a viable treatment with good long-term results. Our case shares with previous cases reported the deficient acetabular coverage and the lack of capsule repair. Nonetheless, to our knowledge, this is the first time that a case of a late presenting post-arthroscopic instability is described. Recent evidence highlights the role of primary capsule repair [3,10] and the identification of patients at risk of developing instability after hip arthroscopy [2,3,7,10,12].

## Consent

Written informed consent was obtained from the patient for publication of this case report and accompanying images. A copy of the written consent is available for review by the Editor-in-Chief of this journal on request.

## Ethical approval

The final version of this manuscript was submitted to the Fundación Valle del Lili ethics committee and approved for submission via Act Num. 01, 2023, Report case No. 636, IRB Approval No. 712-2022 on December 26, 2022.

## Funding

This research did not receive any specific grant from funding agencies in the public, commercial, or not-for-profit sectors.

## Author contribution

Milán Fernando Zárate Leal: Conceptualization, investigation, writing – original draft, review & editing.

María Bautista: investigation, writing – original draft, visualization, writing – review & editing.

Alfredo Sánchez-Vergel: Conceptualization, investigation, resources, supervision, writing – review & editing.

## Guarantor

Milán Fernando Zárate Leal

María Bautista

Alfredo Sánchez-Vergel

## Research registration number

NA.

## Conflict of interest statement

Zarate: none.

Bautista: none.

Sanchez-Vergel: paid speaker for DePuy Johnson & Johnson, Medacta.
